# Peripheral Immune Cell Populations Associated with Cognitive Deficits and Negative Symptoms of Treatment-Resistant Schizophrenia

**DOI:** 10.1371/journal.pone.0155631

**Published:** 2016-05-31

**Authors:** Emilio Fernandez-Egea, Petra E. Vértes, Shaun M. Flint, Lorinda Turner, Syed Mustafa, Alex Hatton, Kenneth G. C. Smith, Paul A. Lyons, Edward T. Bullmore

**Affiliations:** 1 NIHR Cambridge Biomedical Research Centre, Cambridge University Hospitals NHS Foundation Trust and the University of Cambridge, Cambridge, United Kingdom; 2 University of Cambridge, Behavioural & Clinical Neuroscience Institute, Department of Psychiatry, Cambridge, United Kingdom; 3 Centro de Investigación Biomedica en Red de Salud Mental (CIBERSAM), G04, Barcelona, Spain; 4 Cambridgeshire & Peterborough NHS Foundation Trust, Cambridge, United Kingdom; 5 Department of Medicine and Cambridge Institute for Medical Research, University of Cambridge, School of Clinical Medicine, Cambridge, United Kingdom; 6 GlaxoSmithKline, ImmunoPsychiatry, Alternative Discovery & Development, Pharmaceutical R&D, Cambridge, United Kingdom; Beth Israel Deaconess Medical Center, Harvard Medical School, UNITED STATES

## Abstract

**Background:**

Hypothetically, psychotic disorders could be caused or conditioned by immunological mechanisms. If so, one might expect there to be peripheral immune system phenotypes that are measurable in blood cells as biomarkers of psychotic states.

**Methods:**

We used multi-parameter flow cytometry of venous blood to quantify and determine the activation state of 73 immune cell subsets for 18 patients with chronic schizophrenia (17 treated with clozapine), and 18 healthy volunteers matched for age, sex, BMI and smoking. We used multivariate methods (partial least squares) to reduce dimensionality and define populations of differentially co-expressed cell counts in the cases compared to controls.

**Results:**

Schizophrenia cases had *increased* relative numbers of NK cells, naïve B cells, CXCR5^+^ memory T cells and classical monocytes; and *decreased* numbers of dendritic cells (DC), HLA-DR^+^ regulatory T-cells (Tregs), and CD4^+^ memory T cells. Likewise, within the patient group, more severe negative and cognitive symptoms were associated with *decreased* relative numbers of dendritic cells, HLA-DR^+^ Tregs, and CD4^+^ memory T cells. Motivated by the importance of central nervous system dopamine signalling for psychosis, we measured dopamine receptor gene expression in separated CD4^+^ cells. Expression of the dopamine D3 (*DRD3*) receptor was significantly increased in clozapine-treated schizophrenia and covaried significantly with differentiated T cell classes in the CD4^+^ lineage.

**Conclusions:**

Peripheral immune cell populations and dopaminergic signalling are disrupted in clozapine-treated schizophrenia. Immuno-phenotypes may provide peripherally accessible and mechanistically specific biomarkers of residual cognitive and negative symptoms in this treatment-resistant subgroup of patients.

## Introduction

Convergent lines of evidence suggest that immune mechanisms are important for pathogenesis of schizophrenia. There is robust genome-wide association of schizophrenia with single nucleotide variants in the MHC region, in other immune-related genes, and at enhancers expressed in immune cell populations [1, 2]. Imaging and post mortem studies have provided evidence of microglial activation in patients [3–5]. Animal models of neurodevelopmental aberration and psychotic-like adult behaviours have been generated by maternal infection during pregnancy [6, 7]. Clinical studies of immune biomarker differences between patients with schizophrenia and healthy volunteers have been reported, providing preliminary evidence for diagnostic abnormalities, for example, in cytokines, cell counts, and peripheral gene expression of immune signalling proteins (8). Transcriptional differences immune genes have mainly reported from microarray analysis of whole blood samples, so that case-control differences in expression cannot be easily disentangled from possible diagnostic differences in the proportions of major immune cell classes (neutrophils, T cells, etc).

It is therefore plausible that evidence of immune-mediated effects on neuronal function, resulting in cognitive impairment or psychotic symptoms, may be found by examining immune cell populations in the peripheral blood. The peripheral immune system and the central nervous system (CNS) functionally communicate with each other by several mechanisms [8, 9], and may even share some of the same key signalling molecules. For example, classical neurotransmitters such as acetylcholine and dopamine are secreted, and their receptors are expressed, by circulating immune cell populations [10, 11].

It therefore makes sense to look for immunological biomarkers of schizophrenia using multi-parameter flow cytometry to measure the cellular composition and activation state of immune cell populations in peripheral blood. Flow cytometry studies of schizophrenia to date have been relatively few and limited in scope [12–16]. There have been several individually robust reports of abnormal immune cell numbers in schizophrenia; but different studies have tended to focus on a limited number of cell types [12] and/or different clinical subgroups, although there is some evidence that first episode and chronic stages of psychotic disorder may be associated with different peripheral immunophenotypes (13).

Here we report detailed flow cytometry-based immunophenotyping of circulating T-cell, B-cell and myeloid populations from 18 patients with chronic schizophrenia (17 of them currently being treated with clozapine) compared to 20 healthy volunteers. The groups were prospectively matched for some major potential confounders and the patients were clinically characterised in terms of psychotic symptoms, cognitive function and clozapine exposure. We used a panel of markers adapted from that proposed by the Human Immune Phenotyping Consortium [17] to measure 73 immune cell populations. These data were analysed using partial least squares (PLS) regression, a multivariate technique to reduce the dimensionality of the immunological and clinical phenotypes [18] and by so doing mitigate multiple comparisons and allow a few key PLS components, or combinations of immune cell populations, predictive of diagnostic or symptom-related clinical states to be identified. Motivated by the robust prior evidence for CNS dopaminergic signalling as a key mechanism for psychotic symptoms and anti-psychotic drugs [19], we measured dopamine receptor expression in separated CD4^+^ T-cells and used PLS to identify the CD4^+^ T-cell subsets most associated with *DRD3* expression.

## Material and Methods

### Design

A cross-sectional case-control design was used to compare a group of patients with chronic schizophrenia and a group of healthy volunteers matched prospectively for mean age, sex, body mass index (BMI) and cigarette smoking; **(Table A in S1 File)**. The study was ethically approved (Health Research Authority NRES Committee East of England–Cambridge South—REC 12/EE/0467). All participants gave informed consent in writing and the consent procedure included a minimum 24 hour period between initial assessment and formal recruitment to allow participants sufficient time to assess the information provided and make a decision. None of the patients were subject to sections of the UK—Mental Health Act.

### Sample

We recruited 20 patients aged 18–50 years old with a primary diagnosis of chronic schizophrenia, diagnosed by a consultant psychiatrist (EFE) according to F20.X ICD-10 standards [20]. Patients were recruited principally from a specialist treatment-resistant psychosis service provided by the Cambridgeshire & Peterborough NHS Foundation Trust, UK and satisfied formal criteria for treatment resistant schizophrenia [21]. Most patients in this service are being monitored during clozapine treatment for psychotic symptoms that have not responded to first-line anti-psychotic drugs. 20 healthy volunteers matched by age, gender, smoking status and body mass index were prospectively recruited from the NIHR Cambridge BioResource [22]. Exclusion criteria for both groups included major medical disorders, included allergies and immune illness; current illegal drug use (assessed by the Cannabis Experience Questionnaire [23]); or current treatment with any immunomodulatory or anti-inflammatory drugs. The study was conducted between April 2013 and May 2014 and cases and controls were recruited in parallel throughout, controlling approximately for seasonal effects on peripheral immune markers.

### Clinical and cognitive phenotypes

We collected demographic data, medical and cigarette smoking history by structured interview of all participants. The Brief Assessment of Cognition for Schizophrenia (BACS) [24] was used to summarise overall cognitive performance by a composite Z-score for each subject. Psychotic symptom severity was assessed by an experienced psychiatrist completing the Clinical Global Impression for Schizophrenia (CGI-S) [25]. Types and doses of current psychotropic medication, and the most recent plasma concentrations of clozapine, were ascertained by review of the patients’ medical records.

### Peripheral blood immunophenotyping

Each participant provided a 100 mL sample of peripheral venous blood, All samples were extracted between 9 and 10:30 a.m. and no fasting was required. Immuno-phenotyping was conducted as described in **S1 File** using the antibody panel described in **Table B, Table C and Table D in S1 File** [17]. The gating strategy is outlined in **Fig A in S1 File**. Leukocyte populations were expressed both in relative terms as a proportion of a parent population, and as an absolute concentration (cells/mm^3^); **Fig B in S1 File**. Absolute concentrations were estimated from count data obtained concurrently from a TruCount analysis, run according to manufacturer’s instructions (BD Immunocytpmetry Systems). Cytometry data from 4 participants (2 from each group) did not pass quality control criteria and were excluded from further analysis. Summary statistics for the absolute number of cells counted for each population in the sample are given in **Table E in S1 File**.

### Statistical analysis of cytometry data

We planned *a priori* to test two descriptive hypotheses: i) that there are significant case-control differences between patients with schizophrenia and healthy volunteers in the proportions of peripheral immune cells; ii), that there are significant associations between immunological phenotypes and measures of cognitive and clinical symptoms in the patients. We tested both these hypotheses primarily using the multivariate method of partial least squares (PLS).

Partial least squares is useful for identifying associations between a set of response variables and a set of predictor variables, especially when the number of predictor variables exceeds the number of observations, and when the predictor variables are highly interdependent or multi-collinear [18]. Here, the predictor variables were leukocyte populations expressed in relative terms and measured by flow cytometry. In the first analysis, including schizophrenia cases and healthy volunteers, the response variable was the diagnostic status of each participant (case = 1, control = 0). This yielded a number of PLS components, representing the weighted sums of leukocyte populations most strongly associated with diagnosis. In the second PLS analysis, using data only from the patients, the response variable matrix comprised positive, negative and overall psychotic symptom ratings (CGI-S) and cognitive test scores (BACS), yielding PLS components that could be interpreted as the combination of scaled leukocyte populations most strongly associated with individual differences in cognitive function and psychotic symptoms within the patient group; see **S1 File**.

To address potentially confounding effects on the case-control comparison, the two groups were prospectively matched for sex, age, BMI, smoking and zero illegal drug use; **Table A in S1 File**. Prior to the second PLS analysis, the normalised confounding variables above, and clozapine plasma levels (mg/L), were regressed from each response and predictor variable. We used permutation testing to assess the statistical significance of both PLS analyses, and bootstrapping to identify the specific predictor variables (immune cell counts) which made the strongest contributions to explaining the variance in the response variables; see **SuppIementary Information**.

### Dopamine receptor gene expression

The expression levels of all 5 dopamine receptor genes were measured in separated CD4^+^ T-cells by quantitative polymerase chain reaction (PCR). Only *DRD3* and *DRD4* expression levels were within the range of the assay; after missing samples were excluded due to low RNA yields there were 16 subjects per group for *DRD3* and 15 patients and 16 healthy controls for *DRD4*. We tested the case-control difference by a *t*-test for each gene. We also used PLS to identify which of the CD4^+^ T-cell subsets were most strongly associated with CD4^+^ T-cell *DRD3* expression.

## Results

### Sample characteristics

We analysed complete data on 18 patients with treatment resistant schizophrenia (mean duration = 18 years) and 18 healthy volunteers. The two groups were matched in terms of age (mean = 40 years), gender (16/18 male), BMI (mean = 30.5 kg/m^2^) and smoking habits (1/3 current smokers in each group with mean cigarettes/day = 6); **Table A in S1 File**. The patients had significant cognitive impairment (BACS *Z*-score = -1.52 compared with *Z* ~ 0 in healthy volunteers; P = 4 x 10^−5^; Table A in S1 File) and residual positive, negative and overall psychotic symptoms, with a mean score of 3/7 on the CGI questionnaire scales; see **Table A in S1 File**. Most (17/18) patients were receiving clozapine treatment (mean dose = 316 mg/day) and 1 patient was treated with risperidone (6 mg/day).

#### Immunophenotype of schizophrenia

**Fig 1:**
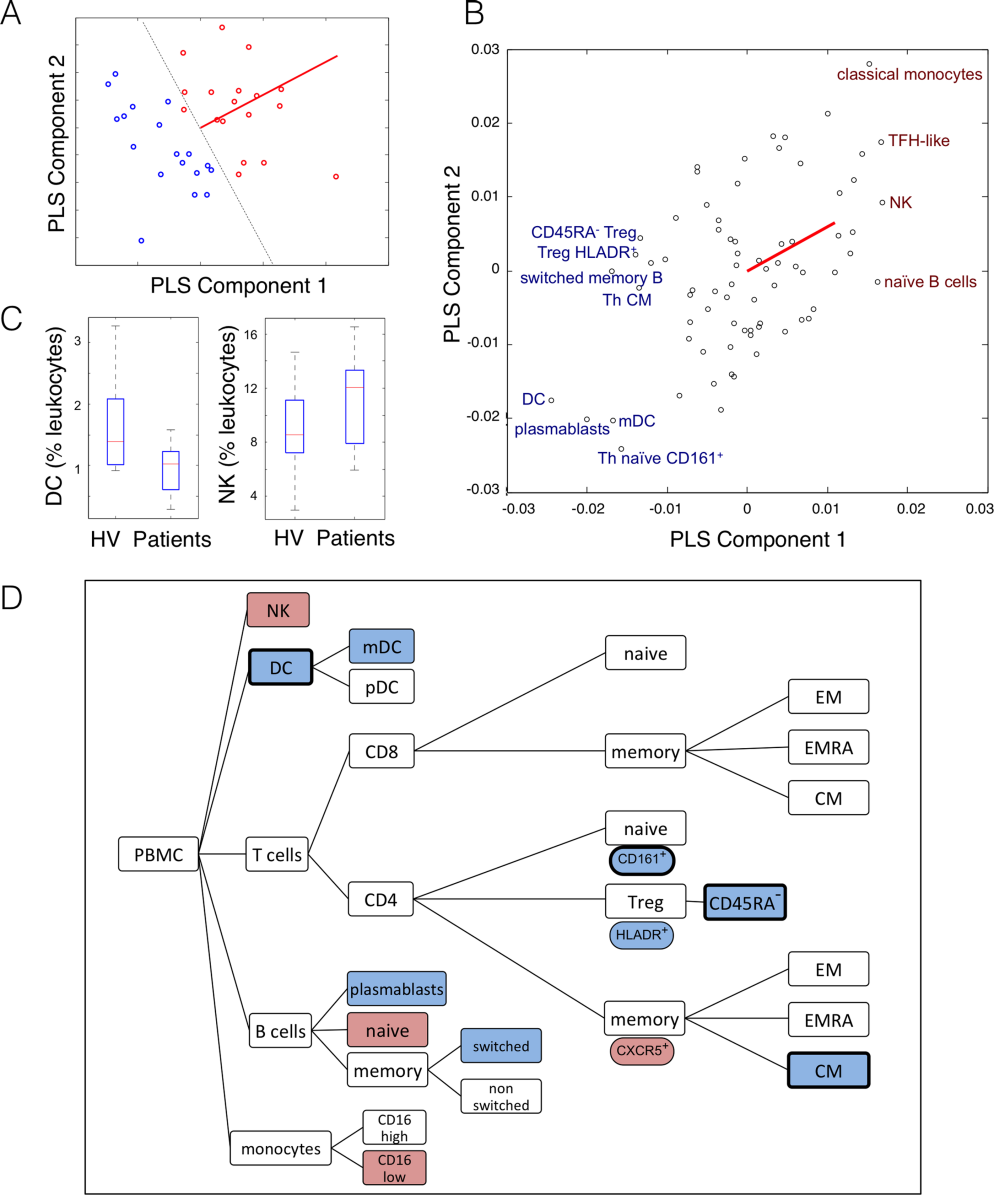
Immune cell populations associated with the case-control difference: patients with clozapine-treated schizophrenia versus healthy volunteers. (A) Separation of participants based on partial least squares (PLS) analysis of the diagnostic response variable: *x*-axis, each subject’s scores on the first PLS component; *y*-axis, participant scores on the second PLS component; healthy volunteers = blue points, people with schizophrenia = red points. The diagnostic boundary (broken black line) correctly classifies 97% of all participants; the loading of the binary predictor variable (red line orthogonal to the diagnostic boundary) indicates that diagnosis of schizophrenia is associated with increased scores on both the first two components. (B) Scatter plot of the weights of individual predictor variables (immune cell counts; black points) on the top two PLS components; the diagnostic loading vector is again represented (red line). Text labels identify cell classes that had the highest positive weights (red text) or highest negative weights (blue text) on one or both of the first two PLS components (based on a bootstrapping procedure). Using the threshold that *Z*-normalised absolute weight must be greater than 3, we have highlighted 5 immune cell populations that have positive weights, and 8 populations that have negative weights, in people with schizophrenia. (C) The weighting of selected cell populations on PLS components relates directly to their relative cell counts in people with schizophrenia and healthy volunteers. Negatively weighted cell populations, like dendritic cells, were decreased in patients; whereas positively weighted cells, like NK cells, were increased in patients. See **Table E in S1 File** for weights on the first two PLS components, for all immune cell classes with *Z*-normalised absolute weight greater than 1; and **Table G in S1 File** for absolute cell counts of major leukocyte populations in both groups. (D) Cytometry tree diagram (simplified) showing all cell classes that were strongly positively weighted (4 red boxes), or strongly negatively weighted (8 blue boxes), on either component in people with schizophrenia. Cell classes that were equally strongly negatively weighted on the first component of the second, symptom-related PLS analysis (**Fig 2**) are highlighted by a black border.

We first used PLS analysis to identify the immune cell populations most strongly predictive of diagnosis (schizophrenia versus healthy volunteer). The first two PLS components together explained 72% of the variance in the response (diagnosis) and could be used to correctly classify 97% of the subjects in the sample (bootstrap classification accuracy ~ 80%); see **Fig 1** and **Fig C in S1 File**. This goodness-of-fit of a low dimensional (2 component) PLS solution to the much higher dimensional relationship between 73 immune cell populations (or predictor variables) and 1 binary diagnostic code (or response variable), was significantly improbable under the null hypothesis that diagnosis is unrelated to the peripheral leukocyte populations (permutation test, *P* = 0.045).

The first PLS component (PLS1) accounted for 53% of diagnostic variance and was significantly negatively correlated with BACS scores (Pearson’s correlation coefficient, *r* = -0.46; *P* = 0.004), i.e., subjects with low BACS scores—indicating cognitive impairment—had positive PLS1 scores. The second PLS component (PLS2) accounted for 19% of diagnostic variance and it was not correlated with BACS scores (*r* = -0.07, *P* = 0.7). Scores on both components were not correlated with age, smoking, or BMI over all subjects; nor with clozapine plasma concentration in the schizophrenia group; see **Table 1**.

**Table 1:** First two PLS component scores for diagnostic and for cognitive and psychotic symptom-related PLS analyses. In both cases, PLS component scores were significantly correlated with cognitive (BACS) or symptom (CGI) scores, but not with confounds (age, smoking, BMI).

	Diagnostic PLS		Symptom-Related PLS	
	*First Component*	*Second Component*	*First Component*	*Second Component*
Age	r = -0.06 P = 0.7	r = 0.21 P = 0.2	*r* = 10^−17^ *P* = 1	*r* = 10^−16^ *P* = 1
Smoking	r = 0.09 P = 0.6	r = -0.01 P = 0.9	*r* = 10^−17^ *P* = 1	*r* = 10^−16^ *P* = 1
BMI	r = 0.01 P = 0.9	r = 0.13 P = 0.4	*r* = 10^−17^ *P* = 1	*r* = 10^−17^ *P* = 1
Clozapine Plasma Levels	r = 0.03 P = 0.9	r = -0.17 P = 0.5	*r* = 10^−17^ *P* = 1	*r* = 10^−17^ *P* = 1
BACS	**r = -0.46 P = 0.004**	r = -0.07 P = 0.7	***r* = -0.52 *P* = 0.05**	*r* = 0.24 *P* = 0.4
CGI-Overall			***r* = 0.68 *P* = 0.005**	*r* = 0.36 *P* = 0.2
CGI-Positive			*r* = 0.29 *P* = 0.3	*r* = 0.48 *P* = 0.07
CGI-Negative			***r* = 0.70 *P* = 0.003**	*r* = -0.24 *P* = 0.4

For PLS1, the most positively weighted cell types were NK cells, naïve B-cells, and CXCR5^+^ memory (i.e. CD45RA^-^) CD4^+^ T-cells; whereas the most negatively weighted cell types included circulating dendritic cells (DC), central memory (i.e. CCR7^+^CD45RA^-^) CD4^+^ T-cells, HLA-DR^+^ Tregs, CD45RA- Tregs, CD4^+^ CD161^+^ naïve T cells and ‘switched memory’ (i.e. CD27^+^IgD^-^) B cells. For PLS2, the most positively weighted cell types were CD16^low^ monocytes; see **Fig 1** and **Table D in S1 File.** Positively weighted populations were relatively increased in patients compared to volunteers; whereas negatively weighted populations were relatively decreased in patients; **Fig 1**. We explored whether populations weighted significantly on a PLS component were also different in absolute numbers, finding that circulating DCs were also fewer in absolute terms in the schizophrenia group (0.03 ± 0.01 x 10^9^ cells/L) compared to healthy volunteers (0.06 ± 0.05 x 10^9^ cells/L; *t*-test, *P* = 0.008); see **Fig 1** and **Table G in S1 File**.

#### Immunophenotype of cognitive impairment and negative symptoms in schizophrenia

In the second PLS analysis, the response variable was a matrix of 4 clinical and cognitive scores (BACS composite score; overall (O), positive (P) and negative (N) symptom scores on the CGI-S) for each of 18 patients with schizophrenia. The BACS score was negatively correlated with overall symptom severity (measured by CGI-O; *r* = -0.62) and with negative symptom severity (measured by CGI-N; *r* = -0.72); i.e., patients with greater cognitive impairment tended to have more severe negative symptoms or a combined cognitive/negative deficit. Psychotic symptom severity and cognitive function were not significantly correlated with age, BMI, cigarette smoking, or plasma concentration of clozapine; see **Table H in S1 File** for details.

**Fig 2:**
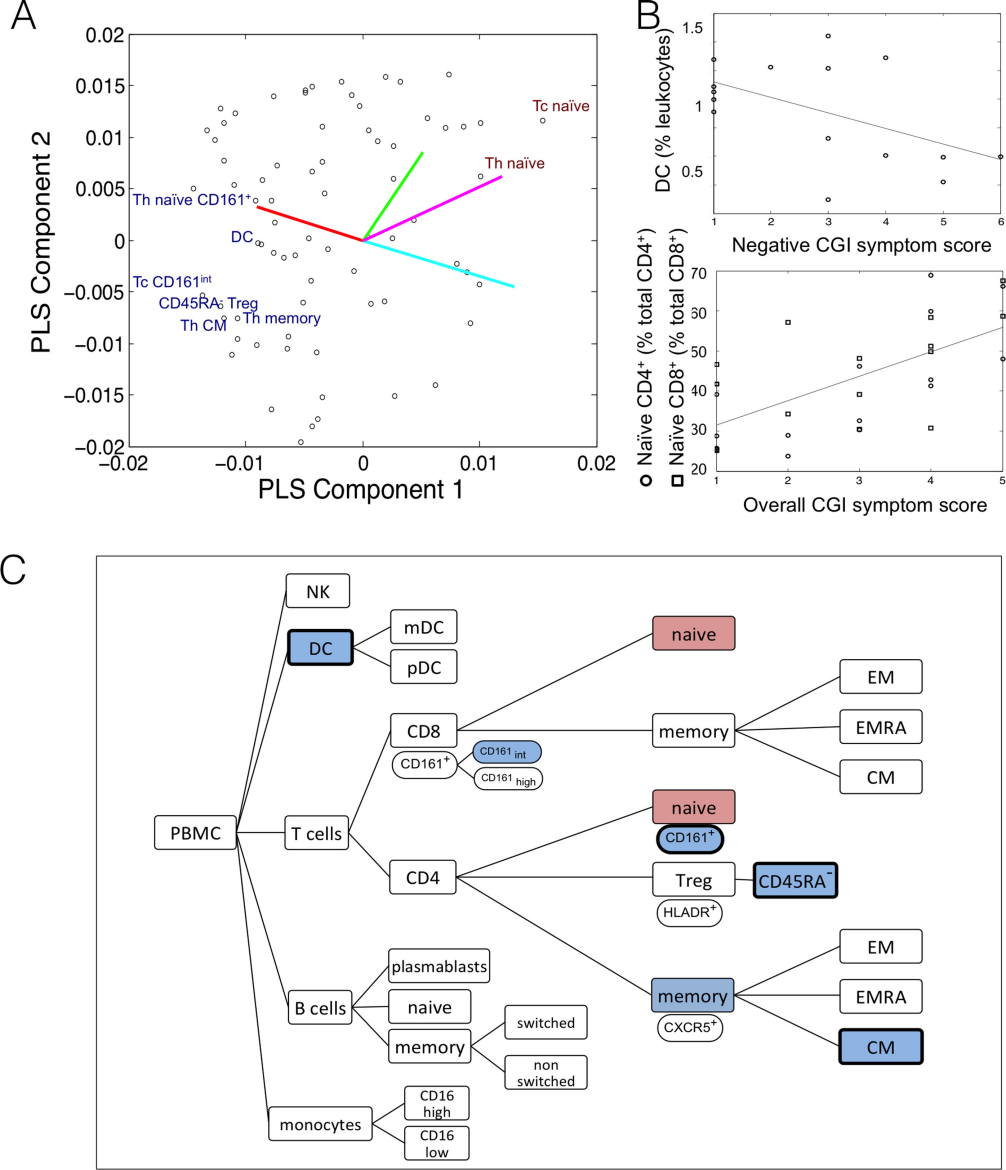
Immune cell populations associated with cognitive deficit and negative symptom severity in patients with clozapine-treated schizophrenia. (A) Scatter plot of the weight of individual predictor variables (immune cell populations) on the first two components of the PLS analysis. The loading vectors for each response variable are also shown: cognitive scores (BACS; red line), negative symptom scores (CGI-N; cyan line), positive symptom scores (CGI-P; green line) and overall psychotic symptom scores (CGI-O; magenta line). The first component is essentially a contrast between high BACS scores (indicating good cognitive function) and high CGI-N scores (indicating severe negative symptoms). Text labels identify cell classes that had the highest positive weights (red text) or highest negative weights (blue text) on the first PLS component (based on a bootstrapping procedure). (B) The weighting of selected cell populations on the first component predicts how the corresponding cell counts are correlated with cognitive and symptom severity scores. Negatively weighted cell classes, like dendritic cells, Treg and CD4^+^ memory T cells, have reduced relative counts in patients with more severe negative symptoms (CGI-N) and more impaired cognitive function (BACS); whereas positively weighted cells, like naïve CD4^+^ and CD8^+^ cells, have increased relative counts in more severely symptomatic patients. (C) Using the threshold that bootstrapped *Z*-normalised absolute weight must be greater than 3, we have highlighted 2 classes that have positive weights, and 6 classes that have negative weights, on the first PLS component. Cell classes that were equally strongly negatively weighted on the first component of the first, diagnostic PLS analysis (**Fig 1**) are highlighted by a black border. See **Table I** in S1 File for weights on the first two PLS components, for all immune cell classes with *Z*-normalised absolute weight greater than 1.

The first two PLS components explained 63% of the variance in cognitive and symptom severity scores: 47% by PLS1 and 16% by PLS2; the goodness-of-fit was significant by permutation testing (*P* = 0.03). The first PLS component was negatively correlated with BACS scores (*r* = -0.52, *P* = 0.05) and positively correlated with CGI-O (*r* = 0.68, *P* = 0.005) and with CGI-N scores (*r* = 0.70, *P* = 0.003); **Fig 2**. The second PLS component was positively, but not significantly, correlated with CGI-P scores (*r* = 0.48, *P* = 0.07). In view of the lack of robust association between PLS2 and any clinical variable, we will refrain from interpreting this component as a candidate immunophenotype of psychotic symptoms; see **Table I in S1 File** for details. The correlations between PLS1 scores and age, BMI, smoking and clozapine concentration were all near zero; **Table 1**.

For PLS1, the most positively weighted populations were naïve CD4^+^ and CD8^+^ T cells; whereas the most negatively weighted populations included DCs, CD45RA^-^ Treg cells, CD45RA^-^ CD4^+^ T cells and CD161^+^ naïve CD4^+^ T cells; see **Fig 2** and **Table I in S1 File**. In other words, the T-cell immunophenotype of a patient with poor cognitive function and/or high negative symptom scores was characterised by a proportional shift toward naïve T-cells from antigen experienced (i.e. CD45RA^-^) CD4^+^ T-cell and Treg populations; see **Fig 2B**. We note that a number of the immune cell populations significant in this analysis were also proportionally reduced in the case-control PLS; these consistently highly weighted cell classes are highlighted in **Figs 1** and **2**.

### Dopamine receptor gene expression

Motivated by the evidence for dopaminergic signalling in the CNS as a key mechanism for pathogenesis and pharmacological treatment of schizophrenia, we tested the mechanistic hypothesis that dopamine receptor expression may also be altered in separated CD4^+^ T-cells, and that this may be correlated with observed alterations in T-cell subpopulations.

**Fig 3:**
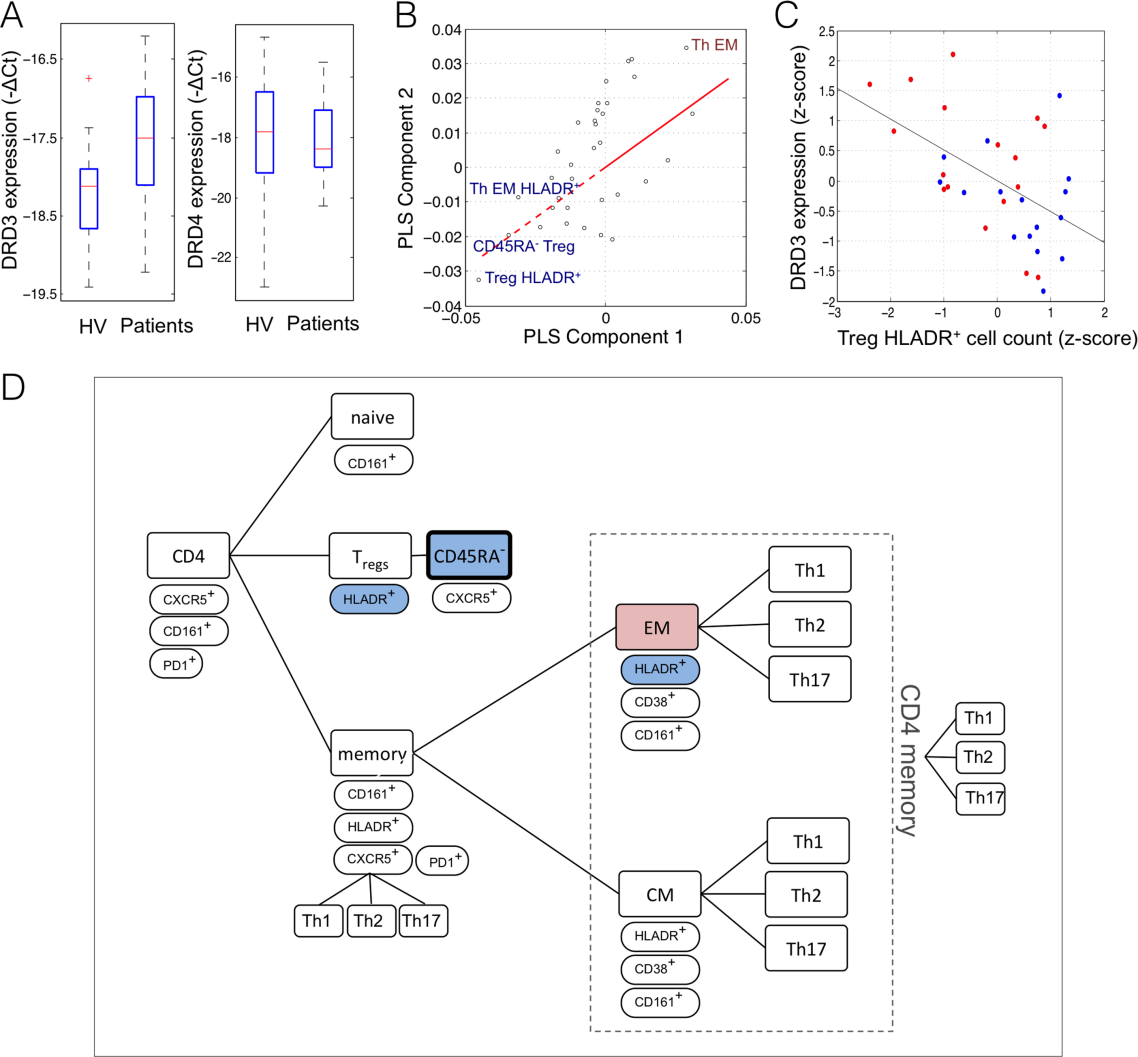
Dopamine D3 receptor gene expression on peripheral T cells. (A) Boxplots showing increased *DRD3* receptor gene expression by CD4^+^ T cells in patients with schizophrenia compared to healthy volunteers (HV) (*t* = -2.2, df = 30, *P* = 0.035), with no significant group-difference in *DRD4* expression. (B) Scatter plot of the weight of individual predictor variables (immune cell populations) on the first two components of the PLS analysis. The loading vector for the response variable (*DRD3* expression) is shown with the solid (dashed) red line indicating increased (decreased) *DRD3* expression. Text labels identify four cell classes that had the highest positive weights (red text) or highest negative weights (blue text) on the first two PLS components (|Z|>3; bootstrapping). The cell types most strongly predictive of *DRD3* expression were HLADR^+^ Treg and memory Treg. (C) Scatterplot shows the negative correlation (*r* = -0.53, df = 30, *P* = 0.002) between *DRD3* receptor expression and HLADR^+^ Treg relative cell counts; red = cases, blue = controls. (D) Hierarchy of the 34 CD4^+^ sub-classes included in the PLS analysis, highlighting the 4 cell classes most strongly related to CD4^+^ *DRD3* expression. Cell classes that were equally strongly negatively weighted in the case-control and within-group PLS analyses (**Figs 1 and 2**) are highlighted in bold.

Dopamine D3 receptor expression (*DRD3)* was detected in CD4^+^ T-cells and was found to be significantly increased in the clozapine-treated schizophrenia group (*t* = -2.2, df = 30, *P* = 0.035), but there was no significant difference in *DRD4* expression; **Fig 3**. *DRD3* expression levels were not significantly correlated with symptom scores (*r* < 0.1 in all, df = 14, *P* > 0.67) or with clozapine exposures in the patient group (*r* = -0.3, df = 12, *P* = 0.3). PLS analysis identified a set of CD4^+^ T-cell subsets that were predictive of CD4^+^ *DRD3* expression (permutation test, *P* = 0.03). Reductions in HLA-DR^+^ and CD45RA^-^ Tregs were strongly correlated with higher *DRD3* expression; **Fig 3**. This is consistent with our findings in **Fig 1** showing reduced counts for both these cell types in patients and with **Fig 3** showing that patients tend to have higher *DRD3* expression levels.

## Discussion

Using multivariate analysis of an extensive set of peripheral immune cell populations measured by flow cytometry in this case-control study, we first identified a subset of leukocyte populations that was strongly associated with clozapine-treated schizophrenia. Some of these immune cell populations were proportionally increased in cases, e.g., NK cells, naïve B-cells, and CXCR5^+^ CD45RA^-^ CD4^+^ T-cells and CD16^low^ monocytes; whereas other populations were proportionally reduced in cases, e.g., dendritic cells (DC), central memory (CCR7^+^CD45RA^-^) CD4^+^ T-cells, HLA-DR^+^ Tregs, CD45RA^-^ Tregs, and CD4^+^ CD161^+^ naïve T cells; **Fig 1**.

### Case-control and within-group analyses in relation to clozapine treatment

We prospectively matched groups for potentially confounding effects of age, gender, obesity and cigarette smoking. However, clozapine remains an obvious potential confounder: all but one of the patients was medicated with clozapine at the time of blood sampling, whereas none of the controls was medicated. Antipsychotic drugs, including clozapine, are known to have effects on peripheral immune cells [26–29]. Clozapine in particular is well known to modify the number of circulating myeloid cells in patients; rare but severe episodes of neutropenia are a well-recognised complication of clozapine and the principal reason for regular blood monitoring of clozapine treatment in specialist clinical services. Clozapine is also clinically associated with greater risk of obesity, diabetes mellitus and metabolic syndrome: all of which modify peripheral immune status. Given the almost complete confounding of clozapine treatment and schizophrenia diagnosis in this sample it is not possible to disentangle the extent to which case-control differences are attributable to the effects of the disorder versus the effects of its treatment. The case-control data are probably therefore best regarded as representative of a sub-group of patients with long-standing (negative and cognitive) psychotic symptoms that have not responded to conventional anti-psychotic drug treatment and have therefore been referred to a specialist NHS treatment service administering and monitoring clozapine for patients with otherwise treatment-resistant schizophrenia.

However, the robustness and specificity of our findings are significantly enhanced by our second analysis, exploring individual differences in cognitive impairment and psychotic symptom severity among the patients with schizophrenia. Since all the participants included in this within-group analysis were exposed to clozapine, the confounding effects of medication should be less severe, and could be statistically mitigated by regression on plasma clozapine levels available for most patients. This analysis identified a number of immune cell populations that were significantly associated with cognitive and psychotic symptom severity within the group of clozapine-treated patients: the strongest positive associations were found for naïve CD4^+^ and CD8^+^ T cells; whereas the strongest negative associations included DCs, CD45RA^-^ Tregs, CD4^+^ CD45RA^-^ T cells and CD161^+^ naïve CD4+ T cells; see **Fig 2** and **Table I in S1 File**. Thus a patient with poor cognitive function and/or high negative symptom scores tended to have proportionally fewer CD45RA^-^ Tregs and circulating DCs. Reassuringly, there was substantial overlap between immune cell populations that were strongly weighted in both the case-control analysis of diagnosis and in this within-group analysis of symptom severity in the cases. Alterations in the proportions of circulating DCs, CD45RA^-^ Tregs, CD4^+^ central memory T cells, and CD4^+^ CD161^+^ naïve T cells were consistently implicated as immune markers of psychosis by both PLS analyses.

Some of these findings can be contextualised by previous cytometry studies of schizophrenia [12] and some are unprecedented. Increased NK cell [13, 30] and monocyte [14, 31, 32] counts have been reported in several prior cytometry studies of schizophrenia, are suggestive of innate immune system activation peripherally, and would be consistent with PET imaging and post-mortem studies showing increased microglial activation in schizophrenia [3, 4]. To our knowledge, this is the first report of reduced circulating DC numbers in schizophrenia. Similarly, we found reduced proportions of HLADR^+^ Tregs and CD45RA^-^ Tregs: these two markers are overlapping and identify a regulatory T-cell population with a more activated phenotype. These results confirm and extend the one prior study of circulating Tregs in schizophrenia, which found reduced numbers overall [14] in the subgroup with poorer outcome. The prior literature on other T cell subsets in schizophrenia includes repeated observations of an increased proportion of naïve T cells relative to CD45RA^-^ CD4^+^ T-cells [33, 34]. We extend this observation by showing that the proportion of naive T-cells increased with increasing overall symptom severity, with a concomitant reduction in antigen experienced (CD45RA^-^) CD4^+^ T-cells. Thus our results are consistent with prior studies [12]; but we have measured many immune cell subsets for the first time in relation to psychosis. Indeed some of the populations we have measured, such as CD161^+^ naïve CD4^+^ T cells, have not yet been well characterised even in normal populations [35].

### Mechanistic interpretation

It is intriguing to speculate how these findings of abnormal circulating innate and adaptive immune cell populations in clozapine-treated patients could be linked to CNS mechanisms for psychotic symptoms and their drug treatment. It is known that dopaminergic signalling in the CNS is both normally important for cognitive function [36, 37] and abnormally perturbed in schizophrenia [38]; and that dopamine D2 receptor antagonists are generally effective anti-psychotic drugs [39]. It is increasingly recognised that dopamine signalling also plays a role in the peripheral immune system. Dendritic cells express all five sub-types of dopamine receptors and can synthesise and secrete dopamine [40]. Similarly, T cells have reliably been shown to express dopamine receptors *DRD3-5*, however the expression of *DRD1* and *DRD2* remains contentious [41, 42]. Dopaminergic signalling modulates or conditions the outcome of immune synapse formation between antigen presenting cells and naïve T cells [40]. Some early reports have suggested that dopaminergic signalling in the immune system may be abnormal in schizophrenia [41, 43–45], in Parkinson’s disease [46] (a neurodegenerative disorder of dopaminergic neurons), and following anti-psychotic drug treatment (risperidone) [43]. For example, schizophrenia has been associated with increased numbers of DRD4 cell surface-expressing CD4^+^ and CD8^+^ T cells [44]; and increased lymphocyte gene expression of *DRD3* [45, 47] (but not *DRD4*) has been reported in three independent prior studies, including in patients not treated with anti-psychotic drugs [47].

We were therefore motivated to test the mechanistically specific hypotheses that there would be abnormal expression of dopamine receptors by peripheral CD4^+^ T cells in clozapine-treated patients with schizophrenia; and that immune cell sub-classes in the CD4^+^ T cell lineage would be associated with individual differences in dopamine receptor expression. Both these predictions were supported. We found significantly higher *DRD3* gene expression in CD4^+^ T cells in schizophrenia; and higher *DRD3* expression was associated with proportionally reduced counts of activated and memory Treg cells. One possible explanation for the association between CD4+ *DRD3* expression levels and counts of CD4^+^ sub-classes of cells is that abnormal dopamine receptor expression on naïve T cells could inhibit their differentiation as a result of immune synaptic contact with antigen presenting cells, leading to relatively reduced numbers of committed Treg cells [48, 49]. However, much remains to be discovered about the interactions between peripheral and central dopamine signalling, in health and disease, before we can be confident about the value of immune cell counts or transcripts as biomarkers of psychotic symptom severity, or of anti-psychotic drug treatment or treatment-resistance.

### Methodological issues

The strengths of this study include the extensive cytometry measurements, the clinical characterization of the patients, and the novel use of partial least squares to reduce dimensionality and mitigate multiple comparisons. The principal limitations include the simple case-control design and modest sample size. The design is potentially vulnerable to uncontrolled differences between the groups in confounding factors, such as exposure to anti-psychotic drugs [50]. We used regression to control the effects of clozapine exposure (and other potential confounds) on immune and cognitive/behavioural variables; and we showed that clozapine exposure was not significantly associated with any of the key PLS components. However, although clozapine concentrations provide a more precise estimate of peripheral exposure than prescribed dose, they are subject to potentially confounding genetic effects on drug metabolism and pharmacokinetics [51]. The pharmacodynamic effects of clozapine or its metabolites on immune function are currently unclear, which further complicates and could undermine the utility of these statistical procedures to control for the effects of clozapine treatment on the within-group analysis. Clearly, it will be important to replicate these results in larger samples, including studies of treatment-naïve patients. Another limitation of the current study is that we did not directly measure central dopaminergic markers, e.g., using positron emission tomography, and we can therefore only speculate how peripheral immune markers might be related to CNS mechanisms known to be important for pathogenesis and treatment of schizophrenia.

## Conclusions

As anticipated by evolving neuroimmunological models of schizophrenia, we conclude that specific combinations of circulating immune cell populations, as measured by flow cytometry, and dopamine receptor expression by these populations, are abnormal in patients with treatment-resistant schizophrenia. Specifically, we highlight the evidence for decreased dendritic cells and committed T or B cells, the evidence for increased NK cells, and the evidence for abnormal dopamine receptor expression on CD4^+^ cells. Further studies of immune cell counts and transcripts in larger and more diverse clinical samples may lead to the validation of peripherally accessible and mechanistically specific biomarkers of psychosis and/or anti-psychotic drug treatment [52].

## Supporting Information

S1 File Sample processing and Partial least squares. Demographic and clinical characteristics of the simple (Table A). Nine and ten colour antibody staining combinations used for flow cytometry (FACS) analysis of immunophenoptypes (Table B). Antibody clones and fluorochrome conjugates used for FACS analysis (Table C). Lineage markers used to determine specific cellular phenotypes (Table D). Descriptive statistics on absolute counts of cell types highlighted in PLS analyses (Table E). Most positively and negatively weighted predictor variables for PLS analysis of diagnostic response variable (Table F). Absolute numbers of cell populations and between-group comparisons (Table G). Correlations of clinical and sociodemographic variables in the schizophrenia group (Table H). Most positively and negatively weighted predictor variables for PLS analysis of symptom severity (Table I). Identification of 73 cell classes by 12-parameter, 10-colour flow cytometry (FACS) (Fig A), Full tree-like cytometry hierarchy showing all 73 cell types and markers measured by FACS (Fig B). Diagnostic classification of subjects by partial least squares (PLS) analysis of the diagnostic response variable (Fig C).
(DOCX)
