# Supplementary material for: Peripheral Immune Cell Populations Associated with Cognitive Deficits and Negative Symptoms of Treatment-Resistant Schizophrenia
Source: PLoS One. 2016 May 31;11(5):e0155631. doi: 10.1371/journal.pone.0155631 (PMC4887013; doi:10.1371/journal.pone.0155631)
Supplement: S1 File — Demographic and clinical characteristics of the simple (Table A). Nine and ten colour antibody staining combinations used for flow cytometry (FACS) analysis of immunophenoptypes (Table B). Antibody clones and fluorochrome conjugates used for FACS analysis (Table C). Lineage markers used to determine specific cellular phenotypes (Table D). Descriptive statistics on absolute counts of cell types highlighted in PLS analyses (Table E). Most positively and negatively weighted predictor variables for PLS analysis of diagnostic response variable (Table F). Absolute numbers of cell populations and between-group comparisons (Table G). Correlations of clinical and sociodemographic variables in the schizophrenia group (Table H). Most positively and negatively weighted predictor variables for PLS analysis of symptom severity (Table I). Identification of 73 cell classes by 12-parameter, 10-colour flow cytometry (FACS) (Fig A), Full tree-like cytometry hierarchy showing all 73 cell types and markers measured by FACS (Fig B). Diagnostic classification of subjects by partial least squares (PLS) analysis of the diagnostic response variable (Fig C). (DOCX) [file pone.0155631.s001.docx]

**Supplementary Information**

**Peripheral immune cell populations associated with cognitive deficits and negative symptoms of chronic schizophrenia**

Emilio Fernandez-Egea*^1,2,6^, Petra E Vértes*^1^, Shaun M Flint*^3^, Lorinda Turner^3^, Syed Mustafa^2^, Alex Hatton^3^, Kenneth Smith^3^, Paul A Lyons^3^, Edward T Bulllmore^1,2,7^.

*These authors have contributed equally to the manuscript.

1. NIHR Cambridge Biomedical Research Centre, Cambridge University Hospitals NHS Foundation Trust and the University of Cambridge, Cambridge UK

2. University of Cambridge, Behavioural & Clinical Neuroscience Institute, Department of Psychiatry, Cambridge UK

3. Centro de Investigación Biomedica en Red de Salud Mental (CIBERSAM), G04. Barcelona, Spain

4. Cambridgeshire & Peterborough NHS Foundation Trust, Cambridge, UK

5 Department of Medicine and Cambridge Institute for Medical Research, University of Cambridge, School of Clinical Medicine, Cambridge UK

6. GlaxoSmithKline, ImmunoPsychiatry, Alternative Discovery & Development, Pharmaceutical R&D, Cambridge and Stevenage, UK

Supplementary Text

**Sample processing**, pages 3-4

**Partial least squares,** pages 5-6

Supplementary Tables

**Table A.** Demographic and clinical characteristics of the sample.

**Table B.** Nine and ten colour antibody staining combinations used for flow cytometry (FACS) analysis of immunophenoptypes.

**Table C.** Antibody clones and fluorochrome conjugates used for FACS analysis.

**Table D: Lineage markers used to determine specific cellular phenotypes.**

**Table E**. Descriptive statistics on absolute counts of cell types highlighted in PLS analyses.

**Table F.** Most positively and negatively weighted predictor variables for PLS analysis of diagnostic response variable.

**Table G.** Absolute numbers of cell populations and between-group comparisons.

**Table H.** Correlations of clinical and sociodemographic variables in the schizophrenia group.

**Table I.** Most positively and negatively weighted predictor variables for PLS analysis of symptom severity.

Supplementary Figures

**Figure A.** Identification of 73 cell classes by 12-parameter, 10-colour flow cytometry (FACS).

**Figure B.** Full tree-like cytometry hierarchy showing all 73 cell types and markers measured by FACS.

**Figure C.** Diagnostic classification of subjects by partial least squares (PLS) analysis of the diagnostic response variable.

**Sample processing**

Sample collection and PBMC preparation

Each participant provided a single 100mL sample of peripheral venous blood. Samples were taken between 9-10:30 am and participants were not fasted prior to venepuncture. Blood samples were collected into a 3.4mL EDTA tube (for BD TruCount) and 4% citrate (for cell separation and immunophenotyping). Leukocyte subsets were isolated as previously described ^1^. Briefly, peripheral blood mononuclear cells (PBMCs) were isolated by centrifugation over Histopaque 1077 (Sigma) at 700g for 20 minutes at room temperature. PBMCs at the interface were collected, rinsed twice with buffer (1 x PBS, 2 mM EDTA), and suspended in running buffer (1xPBS, 2mM EDTA, 0.5% BSA).

Cell separation

An aliquot of PBMCs (6 million) was used for immunophenotyping. Half of the remaining PBMCs were used for separation of CD4^+^ T-cells. PBMCs were first depleted of monocytes using CD14^+^ microbeads (Miltenyi) and then CD4^+^ T-cells were isolated from the remainder by positive selection using CD4^+^ microbeads (Miltenyi). The purity of the separation was determined by flow cytometry (not shown). Purified CD4^+^ T-cells were lysed using buffer RLT plus (Qiagen) with 10uL β-mercaptoethanol/mL added, then passed through QIAshredders (Qiagen). Lysates were stored at -80°C until required. All samples were processed within 5 hours of venepuncture.

Immunophenotyping

Each of 6 different antibody cocktails (**Table S2**) were used to label 10^6^ PBMCs along with 10 μL Fc block (Miltenyi Biotec) and 0.5 μL LIVEDEAD Blue (Invitrogen) by incubation in a final volume of 100 μl for 20 minutes at 4°C in the dark. After incubation, samples were rinsed with buffer then resuspended in FACS fix (1% formaldehyde, 111mM glucose and 0.02% sodium azide in PBS). They were stored at 4°C until acquired on a 5-laser BD Fortessa flow cytometer (detector configuration as shown in **Table S2**). Single colour compensation tubes (BD CompBeads) were prepared for each of the fluorophores used and acquired at the start of each flow cytometer run.

For direct enumeration of mature T, B and NK cells, whole blood (50 μl) was added to BD TruCount™ tubes along with 20 μl BD Multitest™ 6-colour TBNK reagent (BD Biosciences), and processed as per the manufacturer’s instructions. All samples were analysed within 4 hours of staining.

Samples were gated in FlowJo vX (Miltenyi) according to the schema set out in **Figure S3**. The number of cells falling within each gate was recorded. For analysis, these were expressed either in relative terms (i.e. as a proportion of a ‘parent’ population) or as an absolute concentration, calculated using the concentrations of T, B and NK cells determined using the BD TruCount system.

Cytometry data from 4 participants (2 in each group) did not pass quality control criteria and therefore these subjects were excluded from further analysis.

*RNA extraction and quantitative-PCR gene expression analysis*

RNA was extracted using AllPrep DNA/RNA kits and contaminating genomic DNA was removed by DNAse I treatment (Qiagen). RNA was quantified using a NanoDrop ND-1000 spectrophotometer. RNA (750 ng) was reverse transcribed using the Superscript ® Vilo™ cDNA synthesis kit (Life Technologies) as per the manufacturer’s instructions. Expression levels of the target genes *DRD1, DRD2*, *DRD3, DRD4, DRD5* and an endogenous control gene *β2M* were measured by qPCR (reaction volumes of 10 μl) consisting of cDNA (1/40 final dilution) 150 μM Taqman probes (Hs00265245_s1, Hs00241436_m1, Hs00364455_m1, Hs00609526_m1, Hs00361234_s1 and Hs00984230_m1, respectively) and Taqman® Gene Expression mastermix (all reagents from Life Technologies). qPCR was performed using a LightCycler® 480 (Roche) instrument under the following cycling conditions; 50°C for 2 mins, 95°C for 10 minutes, then 45 cycles of 95°C for 15 seconds and 60°C for 1 minute. ΔCp values were calculated by subtracting the Cp of the target gene from the Cp of the endogenous control gene. Samples were tested in triplicate and excluded from analysis if the standard deviation between triplicates was above 0.5 or if the Cp was above 38 cycles.

**Partial Least Squares (PLS)**

Partial least squares is generally useful for identifying associations between a set of response variables and a set of predictor variables, especially when the number of predictor variables is greater than the number of observations and the predictor variables are highly interdependent or multi-collinear. More formally, consider *X*, an *n* by *p* matrix of predictor variables (relative counts for *p* immune cell types in *n* subjects); and *Y*, an *n* by *m* matrix of response variables (*m* diagnostic codes, psychotic symptom scores or cognitive test scores for *n* subjects). Each column of these matrices is mean centred and normalized (each variable is transformed into Z-scores). Then PLS identifies a set of *k* orthogonal factors called latent variables (or PLS components) t_i_, each of which is a linear combination of the original predictor variables (cell counts). The first PLS component is the combination of predictor variables that has maximum covariance with the response variable or a linear combination of the response variables; the second PLS component is the combination of response variables that is orthogonal to the first latent variable and has the next largest covariance with the response variable(s); and so on [1].

This can be written as:

*T = WX (1)*

with the weight matrix *W* containing the weights of predictor variables that define their linear combinations in PLS components.

This is followed by a regression step where the PLS components *T* obtained from *X* are used to predict the response variables:

*Y* = *TQ^T^*+*E (2)*

The term *TQ*^T^ is the PLS approximation of *Y* (also called “fitted *Y*”); *E* is a residual error term; and *Q* is the so-called loading matrix of *Y.* Individual predictor variables with larger weights are thus more strongly predictive of the response variable(s).

*Statistical significance of PLS results*

To test the statistical significance of PLS results, we used permutation testing (randomly permuting the assignment of the response variables) and calculated *P*-values under the null hypothesis that the goodness of the PLS approximation to the data (as measured by R^2^, the square of the correlation between *Y* and *TQ*^T^) is indistinguishable from the distribution of R^2^ estimated from the correlations between *TQ*^T^ and randomly permuted response variables.

We also used bootstrapping (resampling of subjects, with replacement) to estimate the error on the PLS weights estimated for each cell type. The ratio of the weight of each predictor variable to the bootstrap standard error is approximately equivalent to a Z-score that we can use to rank the predictor variables (**Tables E** and **H**). A cut-off value of |Z|>3 was used to highlight the predictor variables that had weights significantly different from noise ^[2]^.

*Controlling for confounding variables*

Peripheral immune populations are sensitive to sex, age, BMI, and cigarette smoking [3]. Clozapine is also known to be an immunomodulatory drug [4]. To address these potentially confounding effects on the case-control comparison, the two groups were prospectively matched for sex, age, BMI, smoking and illegal drug use. For analyses focussing on symptom severity within the patient group the normalised confounding variables were regressed from each response and predictor variable in the subset (N=15) of patients for whom complete data (including clozapine concentrations) was available. The residuals of these regressions were used for the PLS analysis on symptom severity.

In the case of the PLS for *DRD3* expression in CD4^+^ T-cells all subjects were included in the analysis so clozapine plasma levels were not regressed out.

Table A. **Demographic and clinical characteristics of the sample.** Mean **(**SD) unless otherwise specified

|  | Treatment-resistant schizophrenia (n=18) | Healthy volunteers  (n=18) | *P*-value |
| --- | --- | --- | --- |
| Gender (male, %) | 16 (89) | 16 (89) | 1.00 |
| Age in years | 39.9 (7.53) | 39.9 (8.47) | 1.00 |
| Active Smoking (n,%) | 6 (33) | 6 (33) | 1.00 |
| Cigarettes | 7.6 (12.6) | 5.0 (8.22) | 0.46 |
| Current cannabis use (n) | 0 | 0 | 1.00 |
| Body Mass Index | 31.24 (4.75) | 30.0 (3.62) | 0.33 |
| BACS^1^ – Composite Z-Score | -1.52 (1.36) | 0.09 (1.03) | <0.001 |
| Global Assessment | 70.61 (16.81) | 96.50 (2.75) | <0.001 |
| Duration of illness (years) | 18.1 (7.00; 5-34) |  |  |
| CGI^2^-Overall | 3.17 (1.50, 1-6) |  |  |
| CGI-positive | 2.94 (1.63; 1-6) |  |  |
| CGI-negative | 3.0 (1.57; 1-6) |  |  |
| Clozapine dose (mg)^3^ | 316.2 (135.48) |  |  |
| Clozapine plasma levels (mg/dL)^3^ | 0.41 (0.18) |  |  |
| Norclozapine plasma levels (mg/dL) | 0.33 (0.17) |  |  |

^1^ BACS was used to measure cognitive performance on tests of verbal memory, digit sequencing, motor token, verbal fluency, symbol coding, and the Tower of London test of planning, in all participants. The overall BACS score across cognitive domains was summarised by a composite *Z*-score.

^2^ CGI : Clinical Global Impression. The CGI-S rates 4 symptom domains [positive (P), negative (N), depressive (D) and cognitive (C) symptoms], summarised by an overall (O) severity score. Severity is scored from 1 (normal) to 7 (severe), with 3 considered as the cut-off point for clinically relevant symptoms.

^3^ Clozapine dose and plasma levels are summarized only for patients taking clozapine (N=17).

Table B. **Antibody staining combinations used for flow cytometry (FACS) analysis of immunophenoptypes.** Aliquots of PBMCs were stained with one of six antibody combinations prior to FACS analysis. The laser and detector combination used to detect each antibody is also shown.

|  | | Antibody combinations | | | | | |
| --- | --- | --- | --- | --- | --- | --- | --- |
| Laser | Filter | 1 | 2 | 3 | 4 | 5 | 6 |
| 488nm | **530/30** | CXCR3 | CXCR5 | IgD | CD56 | CXCR3 | CXCR3 |
|  | **695/40** | CD45RA | CD45RA | CD24 | CD123 | CD45RA | CD45RA |
| 561nm | **582/15** | CCR7 | CD25 | IgG | CD116 | CXCR5 | CCR7 |
|  | **780/60** | CCR6 | CCR4 | CD27 | CD11c | CCR6 | CCR6 |
| 640nm | **670/14** | CD38 | CD127 | CD38 | CD16 | PD1 | CD161 |
|  | **780/60** | CD4 | CD4 | CD20 | CD19/CD20 | CD62L | CD4 |
| 405nm | **450/50** | HLA-DR | HLA-DR | CD19 | HLA-DR | CD4 | HLA-DR |
|  | **525/50** |  |  |  | CD3 |  |  |
|  | **605/12** | CD3 | CD3 | CD3 | CD14 | CD3 | CD3 |
|  | **655/8** | CD8 |  |  |  | CD8 | CD8 |
| 355nm | **450/50** | Live-dead | Live-dead | Live-dead | Live-dead | Live-dead | Live-dead |

Table C. **Antibody clones and fluorochrome conjugates used for FACS analysis**.

| Marker | Fluorochrome | Company | Clone | Catalogue Number |
| --- | --- | --- | --- | --- |
| CXCR3 | FITC | R&D | 49801 | FAB160F-100 |
| CXCR5 | AF488 | BD Biosciences | RF8B2 | 558112 |
| IgD | FITC | BD Biosciences | IA6-2 | 555778 |
| CD56 | FITC | eBioscience | MEM188 | 11-0569-42 |
| CD45RA | PerCP-Cy5.5 | eBioscience | HI100 | 45-0458-42 |
| CD24 | PerCP-Cy5.5 | BD Biosciences | ML5 | 561647 |
| CD123 | PerCP-Cy5.5 | eBioscience | 6H6 | 45-1239-42 |
| CCR7 (CD197) | PE | BD Biosciences | 150503 | 560765 |
| CD25 | PE | BD Biosciences | M-A251 | 555432 |
| IgG | PE | BD Biosciences | G18-145 | 555787 |
| CD116 | PE | BD Biosciences | hGMCSFR-M1 | 551373 |
| CXCR5 | PE | R&D | 51505 | FAB190P |
| CCR6 (CD196) | PECy7 | BioLegend | GO34E3 | 343418 |
| CCR4 (CD194) | PECy7 | BD Biosciences | 1G1 | 561034 |
| CD27 | PECy7 | eBioscience | 0323 | 25-0279-42 |
| CD11c | PECy7 | eBioscience | 3.9 | 25-0116-42 |
| CD38 | APC | BD Biosciences | HIT2 | 555462 |
| CD127 (IL-7R) | AF647 | BD Biosciences | HIL-7R-M21 | 558598 |
| CD16 | APC | eBioscience | eBioCD16 | 17-0168-42 |
| CD161 | APC | eBioscience | HP-3910 | 17-1619-42 |
| PD1 (CD279) | APC | eBioscience | J105 | 17-2799-42 |
| CD4 | APC-eF780 | eBioscience | RPA-T4 | 47-0049-42 |
| CD20 | APC-eF780 | eBioscience | 2H7 | 47-0209-42 |
| CD19 | APC-eF780 | eBioscience | HIB19 | 47-0199-42 |
| CD62L | APC-eF780 | eBioscience | DREG-56 | 47-0629-42 |
| HLA-DR | eFluor 450 | eBioscience | L243 | 48-9952-42 |
| CD19 | eFluor 450 | BD Biosciences | HIB19 | 560353 |
| CD4 | Pacific Blue | BioLegend | OKT4 | 317429 |
| CD3 | AmCyan | BD Biosciences | SK7 | 339186 |
| CD3 | NC605 | eBioscience | OKT3 | 93-0037-42 |
| CD14 | NC605 | eBioscience | 61D3 | 93-0149-42 |
| CD8 | NC650 | eBioscience | RPA-T8 | 95-0088-42 |
| Live-Dead | Aqua | Life technologies | | L-23105 |

**Table D: Lineage markers used to determine immune cell populations.**.

| Population Name | Lineage markers |  | Parent population |
| --- | --- | --- | --- |
| **B cell populations** |  |  |  |
| B cells | CD3^-^ CD19^+^ | of | Lymphocytes |
| Naïve B cells | CD3^-^ CD19^+^ IgD^+^ CD27^-^ | of | B cells |
| Switched memory B | CD3^-^ CD19^+^ IgD^-^ CD27^+^ | of | B cells |
| Non-switched memory B | CD3^-^ CD19^+^ IgD^+^ CD27^+^ | of | B cells |
| IgD- CD20+ | IgD^-^ CD20^+^ B cells | of | B cells |
| Transitional B cells | CD3^-^ CD19^+^ IgD^+^ CD27^-^ CD24^high^ CD38^high^ | of | B cells |
| plasmablasts | CD3^-^ CD19^+^ IgD^-^ CD20^+^ CD38^+^ | of | B cells |
| **T cell populations** |  |  |  |
| Double positive T cells | CD3^+^ CD4^+^ CD8^+^ | of | Lymphocytes |
| Double negative T cells | CD3^+^ CD4^-^ CD8^-^ | of | Lymphocytes |
| CD4+ T helper cells (Th) | CD3^+^ CD4^+^ | of | Lymphocytes |
| Th naïve | CD3^+^ CD4^+^ CD45RA^+^ CCR7^+^ | of | CD4+ |
| Th effector memory (EM) | CD3^+^ CD4^+^ CD45RA^-^ CCR7^-^ | of | CD4+ |
| Th EM HLADR^+^ | CD3^+^ CD4^+^ CD45RA^-^ CCR7^-^ HLADR^+^ | of | CD4+ |
| Th EM CD38^+^ | CD3^+^ CD4^+^ CD45RA^-^ CCR7^-^ CD38^+^ | of | CD4+ |
| Th1 EM | CD3^+^ CD4^+^ CD45RA^-^ CCR7^-^ CXCR3^+^ CCR6^-^ | of | CD4+ |
| Th2 EM | CD3^+^ CD4^+^ CD45RA^-^ CCR7^-^ CXCR3^-^ CCR6^-^ | of | CD4+ |
| Th17 EM | CD3^+^ CD4^+^ CD45RA^-^ CCR7^-^ CXCR3^-^ CCR6^+^ | of | CD4+ |
| Th central memory (CM) | CD3^+^ CD4^+^ CD45RA^-^ CCR7^+^ | of | CD4+ |
| Th CM HLADR^+^ | CD3^+^ CD4^+^ CD45RA^-^ CCR7^+^ HLADR^+^ | of | CD4+ |
| Th CM CD38^+^ | CD3^+^ CD4^+^ CD45RA^-^ CCR7^+^ CD38^+^ | of | CD4+ |
| Th1 CM | CD3^+^ CD4^+^ CD45RA^-^ CCR7+ CXCR3^+^ CCR6^-^ | of | CD4+ |
| Th2 CM | CD3^+^ CD4^+^ CD45RA^-^ CCR7^+^ CXCR3^-^ CCR6^-^ | of | CD4+ |
| Th17 CM | CD3^+^ CD4^+^ CD45RA^-^ CCR7^+^ CXCR3^-^ CCR6^+^ | of | CD4+ |
| Th memory | CD3^+^ CD4^+^ CD45RA^-^ | of | CD4+ |
| Th1 memory | CD3^+^ CD4^+^ CD45RA^-^ CXCR3^+^ CCR6- | of | CD4+ |
| Th2 memory | CD3^+^ CD4^+^ CD45RA^-^ CXCR3^-^ CCR6^-^ | of | CD4+ |
| Th17 memory | CD3^+^ CD4^+^ CD45RA^-^ CXCR3^-^ CCR6^+^ | of | CD4+ |
| Th memory HLADR^+^ | CD3^+^ CD4^+^ CD45RA^-^ HLADR^+^ | of | CD4+ |
| CD8+ Cytotoxic T cells (Tc) | CD3^+^ CD8^+^ | of | Lymphocytes |
| Tc naïve | CD3^+^ CD8^+^ CD45RA^+^ CCR7^+^ | of | CD8+ |
| Tc EMRA | CD3^+^ CD8^+^ CD45RA^+^ CCR7^-^ | of | CD8+ |
| Tc memory | CD3^+^ CD8^+^ CD45RA^-^ | of | CD8+ |
| Tc EM | CD3^+^ CD8^+^ CD45RA^-^ CCR7^-^ | of | CD8+ |
| Tc EM HLADR^+^ | CD3^+^ CD8^+^ CD45RA^-^ CCR7^-^ HLADR^+^ | of | CD8+ |
| Tc EM CD38^+^ | CD3^+^ CD8^+^ CD45RA^-^ CCR7^-^ CD38^+^ | of | CD8+ |
| Tc CM | CD3^+^ CD8^+^ CD45RA^-^ CCR7^+^ | of | CD8+ |
| Tc CM HLADR^+^ | CD3^+^ CD8^+^ CD45RA^-^ CCR7^+^ HLADR^+^ | of | CD8+ |
| Tc CM CD38^+^ | CD3^+^ CD8^+^ CD45RA^-^ CCR7^+^ CD38^+^ | of | CD8+ |
| Tc memory HLADR^+^ | CD3^+^ CD8^+^ CD45RA^-^ HLADR^+^ | of | CD8+ |
| Tc CXCR5^+^ | CD3^+^ CD8^+^ CXCR5^+^ | of | CD8+ |
| **Myeloid populations** |  |  |  |
| Dendritic cells (DCs) | CD3^-^ CD19^-^ CD20^-^ CD14^-^ CD56^-^ HLADR^+^ | of | PBMCs |
| mDC | CD3^-^ CD19^-^ CD20^-^ CD14^-^ CD56^-^ HLADR^+^ CD11c^+^ CD123^-^ | of | DCs |
| pDC | CD3^-^ CD19^-^ CD20^-^ CD14^-^ CD56^-^ HLADR^+^ CD11c^-^ CD123^+^ | of | DCs |
| Monocytes | CD3^-^ CD19^-^ CD20^-^ CD14^+^ | of | PBMCs |
| Classical monocytes | CD3^-^ CD19^-^ CD20^-^ CD14^+^ CD16- | of | Monocytes |
| Non-classical monocytes | CD3^-^ CD19^-^ CD20^-^ CD14^+^ CD16 ^high^ | of | Monocytes |
| Natural killer (NK) cells | CD3^-^ CD19^-^ CD20^-^ CD14^-^ CD56^+^ | of | PBMCs |
| NKT cells | CD16^+^ CD56^+^ CD3^+^ | of | PBMCs |
| CD56^high^ NK | CD3^-^ CD19^-^ CD20^-^ CD14^-^ CD56 ^high^ CD16^-^ | of | NK |
| CD16^high^ NK | CD3^-^ CD19^-^ CD20^-^ CD14^-^ CD56^+^ CD16^high^ | of | NK |
| Treg | CD3^+^ CD4^+^ CD25^high^ CD127^low^ | of | CD4+ |
| CD45RA- Treg | CD3^+^ CD4^+^ CD25^high^ CD127 ^low^ CD45RA^-^ | of | Treg |
| Treg HLADR^+^ | CD3^+^ CD4^+^ CD25^high^ CD127 ^low^ HLADR^+^ | of | Treg |
| CD45RA- Treg CXCR5^+^ | CD3^+^ CD4^+^ CD25^high^ CD127 ^low^ CD45RA^-^ CXCR5^+^ | of | Treg |
| **T-follicular helper cell populations** |  |  |  |
| Th CXCR5^+^ | CD3^+^ CD4^+^ CXCR5^+^ | of | CD4+ |
| TFH-like | CD3^+^ CD4^+^CD45RA^-^ CXCR5^+^ | of | CD4+ |
| TFH-like Th1 | CD3^+^ CD4^+^ CD45RA^-^ CXCR3^+^ CCR6^-^ CXCR5^+^ | of | TFH-like |
| TFH-like Th2 | CD3^+^ CD4^+^ CD45RA^-^ CXCR3^-^ CCR6^-^ CXCR5^+^ | of | TFH-like |
| TFH-like Th17 | CD3^+^ CD4^+^ CD45RA^-^ CXCR3^-^ CCR6^+^ CXCR5^+^ | of | TFH-like |
| Th PD1^+^ | CD3^+^ CD4^+^ PD1^+^ | of | CD4+ |
| TFH-like PD1^+^ | CD3^+^ CD4^+^ CD45RA^-^ CXCR5^+^ PD1^+^ | of | TFH-like |
| **CD161+ populations** |  |  |  |
| Th CD161^+^ | CD3^+^ CD4^+^ CD161^+^ | of | CD4+ |
| Th naïve CD161^+^ | CD3^+^ CD4^+^ CD45RA^+^ CCR7^+^ CD161^+^ | of | CD4+ |
| Th EM CD161^+^ | CD3^+^ CD4^+^ CD45RA^-^ CCR7^-^ CD161^+^ | of | CD4+ |
| Th CM CD161^+^ | CD3^+^ CD4^+^ CD45RA^-^ CCR7^+^ CD161^+^ | of | CD4+ |
| Th memory CD161^+^ | CD3^+^ CD4^+^ CD45RA^-^ CD161^+^ | of | CD4+ |
| Tc CD161^+^ | CD3^+^ CD8^+^ CD161^+^ | of | CD8+ |
| Tc CD161^high^ | CD3^+^ CD8^+^ CD161^high^ | of | CD8+ |
| Tc CD161^int^ | CD3^+^ CD8^+^ CD161^int^ | of | CD8+ |
| Tc EMRA CD161^+^ | CD3^+^ CD8^+^ CD45RA^+^ CCR7^-^ CD161^+^ | of | CD8+ |
| Tc EM CD161^+^ | CD3^+^ CD8^+^ CD45RA^-^ CCR7^-^ CD161^+^ | of | CD8+ |
| Tc CM CD161^+^ | CD3^+^ CD8^+^ CD45RA^-^ CCR7^+^ CD161^+^ | of | CD8+ |
| Tc memory CD161^+^ | CD3^+^ CD8^+^ CD45RA^-^ CD161^+^ | of | CD8+ |
| Double negative T cells CD161^+^ CD4-CD- T-cells | CD3^+^ CD4^-^ CD8^-^ CD161^+^ | of | Lymphocytes |

Table E**:** **Descriptive statistics on absolute counts of cell types highlighted in PLS analyses.**This table shows the minimum (min), median and mean (avg) numbers acquired for each cell type of interest across the 36 subjects for which a complete dataset is available. The standard deviation and standard error on the mean (SEM) are also shown. Note that mDC and plasmablasts (highlighted in grey) were rare populations with small numbers of cells acquired in many samples.

Table F: **Most positively and negatively weighted predictor variables for PLS analysis of diagnostic response variable.** Cell types with Z-score values between -1 and 1 are not shown (represented by row containing “…”). Highly significant variables (with |Z|-score>3) are highlighted in red and blue for up and down-regulated cell types.

Table G. **Absolute numbers for major leukocyte populations (cells x 10^9^/L) and P-values for between-group comparisons by Student’s t-test. Mean and standard deviation are presented.**

|  | Schizophrenia (n=18) | Control Group  (n=18) | P value. |
| --- | --- | --- | --- |
| Monocytes | 0.80 (0.26) | 0.96 (0.53) | 0.298 |
| Natural Killer Cells | 0.39 (0.17) | 0.33 (0.16) | 0.365 |
| B-Cells | 0.44 (0.23) | 0.40 (0.22) | 0.643 |
| T-Cells | 1.84 (0.67) | 2.10 (0.96) | 0.349 |
| Dendritic Cells | 0.03 (0.01) | 0.06 (0.05) | 0.008 |

Table H. **Correlations of clinical and demographic variables in schizophrenia group.**

|  | BACS z-score | CGI-P | CGI-N | CGI-O |
| --- | --- | --- | --- | --- |
| Age (in years) | r= .09  p= .75 | r= -.16  p= .57 | r= -.06  p= .84 | r= -.11  p= .70 |
| Smoking (n cigarettes) | r= .14  p= .26 | r= .21  p= .45 | r= -.31  p= .27 | r= .21  p= .44 |
| BMI (Kg/m^2^) | r= .18  p= .51 | r= -.26  p= .35 | r= -.17  p= .54 | r= -.33  p= .22 |
| Clozapine dose | r= -.10  p= .71 | r= .13  p= .63 | r= .03  p= .90 | r= .18  p= 53 |
| Clozapine plasma levels | r= -.33  p= .22 | r= .29  p= .29 | r= .33  p= .23 | r= .28  p= .31 |
| CGI-positive (P) | r= -.153  p= .586 |  |  |  |
| CGI-negative (N) | r= -.72  p= **.002** |  |  |  |
| CGI-overall (O) | r= -.62  p= **.001** |  |  |  |

CGI = Clinical Global Impression. BACS = Brief Assessment of Cognition in Schizophrenia

Table I: **Most positively and negatively weighted predictor variables for PLS analysis of symptom severity.** In the interest of space cell types with Z-score values between -1.5 and 1.5 are not shown (represented by row containing “…”). Highly significant variables (with |Z|-score>3) are highlighted in red and blue for up and down-regulated cell types (respectively).

Figure A: **Flow cytometry gating strategy for 6 separate antibody panels.**

PBMCs were stained with one of six, 10-colour antibody staining combinations (see **Table B**) and analysed by flow cytometry. Panels show the progressive gating strategies to obtain CD4^+^ and CD8^+^ T-cell subsets (A), Tregs (B), B-cell subsets (C), myeloid cell subsets (D), CD161^+^ T-cell subset (E), or circulating T follicular helper-like cell subsets (F).

**
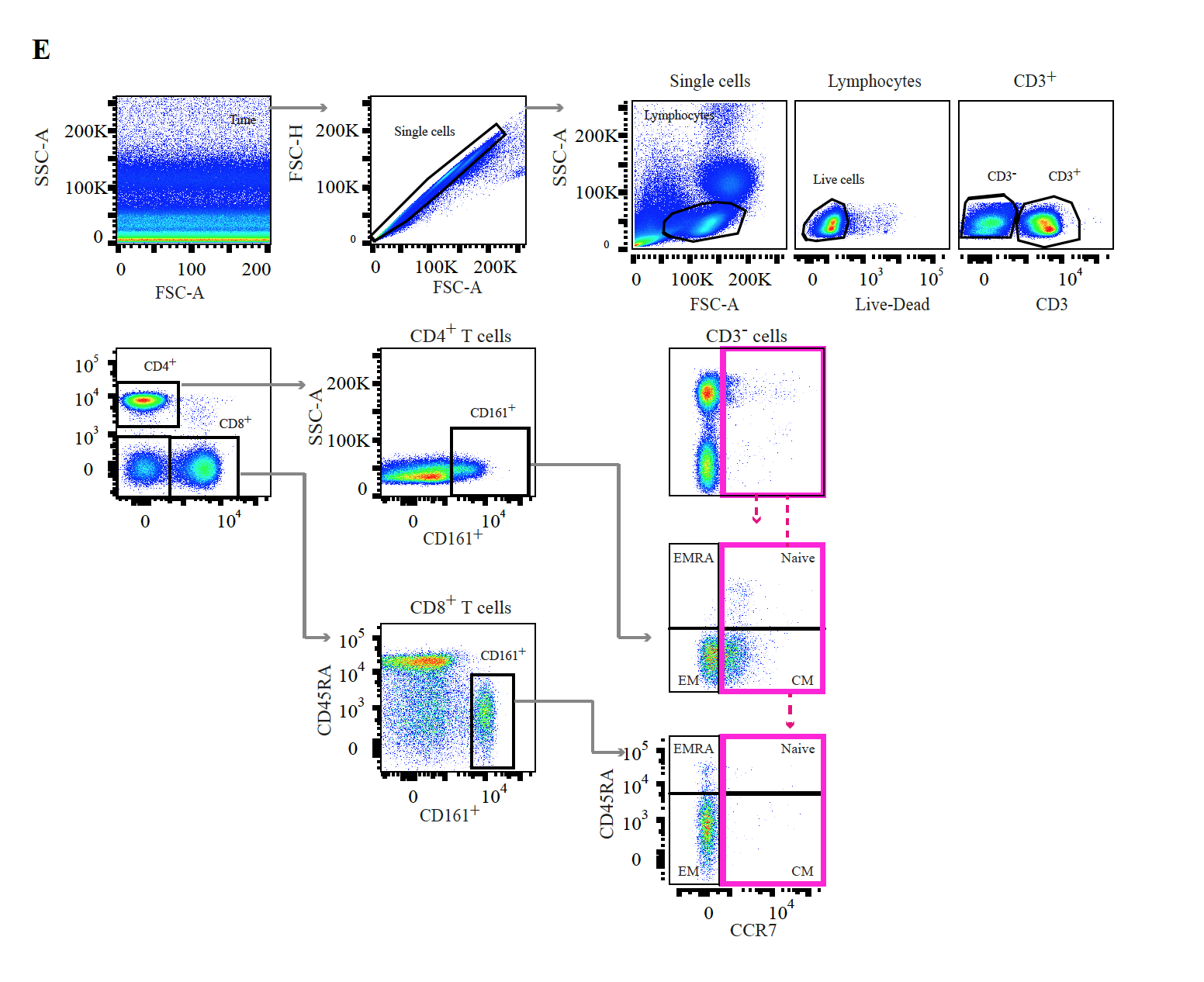
**

Figure B: **Leukocyte populations gated from the flow cytometry data represented as a hierarchy.**

Figure C: **Separation of subjects by partial least squares (PLS) analysis of the diagnostic response variable**. This figure shows the fitted response variable based on the first two PLS components for all 36 subjects. The first 18 subjects were healthy volunteers (blue) and their response variable was zero, while subjects 19 to 36 were patients with schizophrenia with response variable set to one. The dashed line shows the level at which the PLS fit to the response variable is 0.5 (bisecting the interval from zero to one). Subjects whose fitted response falls below this line can be classified as healthy, yielding correct classification in 97% of this dataset.

**References**

1. Abdi H, Williams LJ (2013) Partial least squares methods: partial least squares correlation and partial least square regression. Methods in molecular biology (Clifton, NJ) 930: 549-579.

2. McIntosh AR, Lobaugh NJ (2004) Partial least squares analysis of neuroimaging data: applications and advances. NeuroImage 23 Suppl 1: S250-263.

3. Miller BJ, Gassama B, Sebastian D, Buckley P, Mellor A (2013) Meta-analysis of lymphocytes in schizophrenia: clinical status and antipsychotic effects. Biol Psychiatry 73: 993-999.

4. Røge R, Møller BK, Andersen CR, Correll CU, Nielsen J (2012) Immunomodulatory effects of clozapine and their clinical implications: What have we learned so far? Schizophrenia Research.
